# Titanite petrochronological data across the continental crust section exposed in Val d'Ossola (Ivrea-Verbano Zone, Italy)

**DOI:** 10.1016/j.dib.2024.110804

**Published:** 2024-08-14

**Authors:** Stefania Corvò, Andrew R.C. Kylander-Clark, Antonio Langone

**Affiliations:** a Department of Earth and Environmental Sciences, University of Pavia, Pavia, Italy; b Institute of Geosciences and Earth Resources of Pavia, C.N.R., Pavia, Italy; c Department of Earth Science, University of California, Santa Barbara, United States

**Keywords:** HT metamorphism, Middle/lower continental crust, Calc-silicates, Amphibolites, Petrochronology, U–Pb titanite dating

## Abstract

Titanite-bearing calc-silicates and mafic gneisses, metamorphosed under amphibolite- to granulite-facies conditions, crop out in Val d'Ossola area (Ivrea-Verbano Zone, Italy). The Ivrea-Verbano Zone represents an exhumed section of the pre-Alpine middle to lower continental crust which escaped the Alpine subduction, thus provides a unique opportunity to study continental crustal processes and evolution. Among several samples, three, collected from different locations, were chosen for detailed analyses of titanite. Petrochronology of titanite was performed with Laser ablation split-stream (LASS) technique on petrographic thin sections. Petrochronological results on titanite do not define clear correlations with chemistry except for one sample. Rare earth elements (REE) patterns of titanite from the three samples are apparently different in terms of average concentration (i.e., lower or upper 1000 times CI), shapes and occurrence or absence of Eu negative anomaly. Al/Fe vs ΣLREE and Fe content vs Zr/Y plots show that the studied samples coincide with metamorphic rock field deriving from calc-silicates and mafic protoliths, as previously demonstrated in literature. Any compilations of petrochronological data on titanite from the metamorphic volcano-sedimentary sequence of Val d'Ossola can be found in literature. Therefore, these data represent a new insight on an accessory mineral phase whose significance and scientific interest are rising in the last years. Future studies of the evolution of these kinds of rock, widespread in the high-grade metamorphic basements, will benefit from these data as a term of comparison.

Specifications Table

**Table:** 

Subject	Geochemistry and petrology
Specific subject area	Petrochronological data on titanite within amphibolite and calc-silicates from lower continental crust performed by Laser ablation split-stream (LASS) technique
Type of data	Table, Chart, Graph, Figure
Data collection	Several titanite-bearing rocks from the high-grade amphibolite to granulite metamorphic basement of the Val d'Ossola continental crust section were studied. The most representative ones, consisting of calc-silicates and mafic gneisses, were chosen for the petrochronological analyses. Thin sections and titanites were studied by optical and electron microscopy. Selected titanite grains were analyzed by Laser ablation split-stream (LASS) technique.
Data source location	Data are stored at the Department of Earth and Environmental Science of University of Pavia, Pavia (Italy) GPS coordinates for collected samples: 1° location: Ornavasso, Val d'Ossola, Italy GPS (WGS84) coordinates: Calc-silicate (MV04B) sample 8 °23′ 48′’ E - 45 °58′ 56′’ N 2° location: Anzola, Val d'Ossola, Italy GPS (WGS84) coordinates: Mafic gneiss (AN10) sample 8 °20′ 47′’ E - 45 °59′ 10′’ N Calc-silicate (MV05) sample 8 °21′ 08′’ E - 45 °59′ 08′’ N
Data accessibility	Repository name: Mendeley Data Data identification number: 10.17632/26cbnsz2jn.1 Direct URL to data: https://data.mendeley.com/datasets/26cbnsz2jn/1 Instructions for accessing these data: Corvò, Stefania; Kylander-Clark, Andrew; Langone, Antonio (2024), “Corvò et al. (2024) Data in Brief_U-Pb data by LASS method on Titanite”, Mendeley Data, V1, doi: 10.17632/26cbnsz2jn.1
Related research article	[1] Corvò, S., Maino, M., Piazolo, S., Seno, S., Langone, A., 2022. Role of inherited compositional and structural heterogeneity in shear zone development at mid-low levels of the continental crust (the ASZ; Ivrea-Verbano Zone, Southern Alps). Lithos 106745.

## 1 Value of the Data

• Titanite provides constraints on the genesis, metamorphism and age of the host rock [[2], [3], [4]].
• Petrologists dealing with the evolution of the fossil passive Adriatic margin of the Alpine Tethys can found interesting information on titanite chemistry and ages.
• Regional- scale studies could benefit from these data for comparison with similar rocks.
• Any data about titanite in Val d'Ossola are available in literature.
• Petrological interest for Ti-bearing mineral phases such as titanite is growing in the last years [4].
• Titanite petrochronological data from different compositional rocks are very few in literature.

## 2 Background

Titanite is a useful accessory mineral that, in the last decades, has gained great interest in the petrological community since is considered as a powerful petrochronometer that allow the comprehension of Earth's continental crustal processes [4]. Moreover, titanite is a widespread accessory mineral that crystallizes over a wide range of crustal pressures and temperatures in many rock types, including metamorphosed mafic rocks, calc-silicates as well as felsic calc-alkaline igneous rocks [[3], [4], [5]]. Recently, titanite petrochronology provided unique information about the timing of shear zones [e.g., 2]. Nevertheless, since the strong reactiveness of titanite during metamorphic reactions [3], it can experience and record multi-stage tectono-metamorphic events not always easily distinguishable.

The aim behind this dataset is to provide new petrochronological data of the best preserved (and studied) section of middle to lower continental crust of the world, i.e., the Ivrea-Verbano Zone (IVZ) in the Southern Alps. In particular, we focus on the titanite-bearing rocks outcropping in Val d'Ossola showing progressively higher temperature conditions with increasing crustal depth [6].

Although numerous geochronological and thermochronological data relative to zircon, monazite, mica, hornblende and rutile are available for the IVZ [see 7 for an extensive review]; titanite dating are still rare [2].

## 3 Data Description

### 3.1 Geological setting

**Fig. 1:**
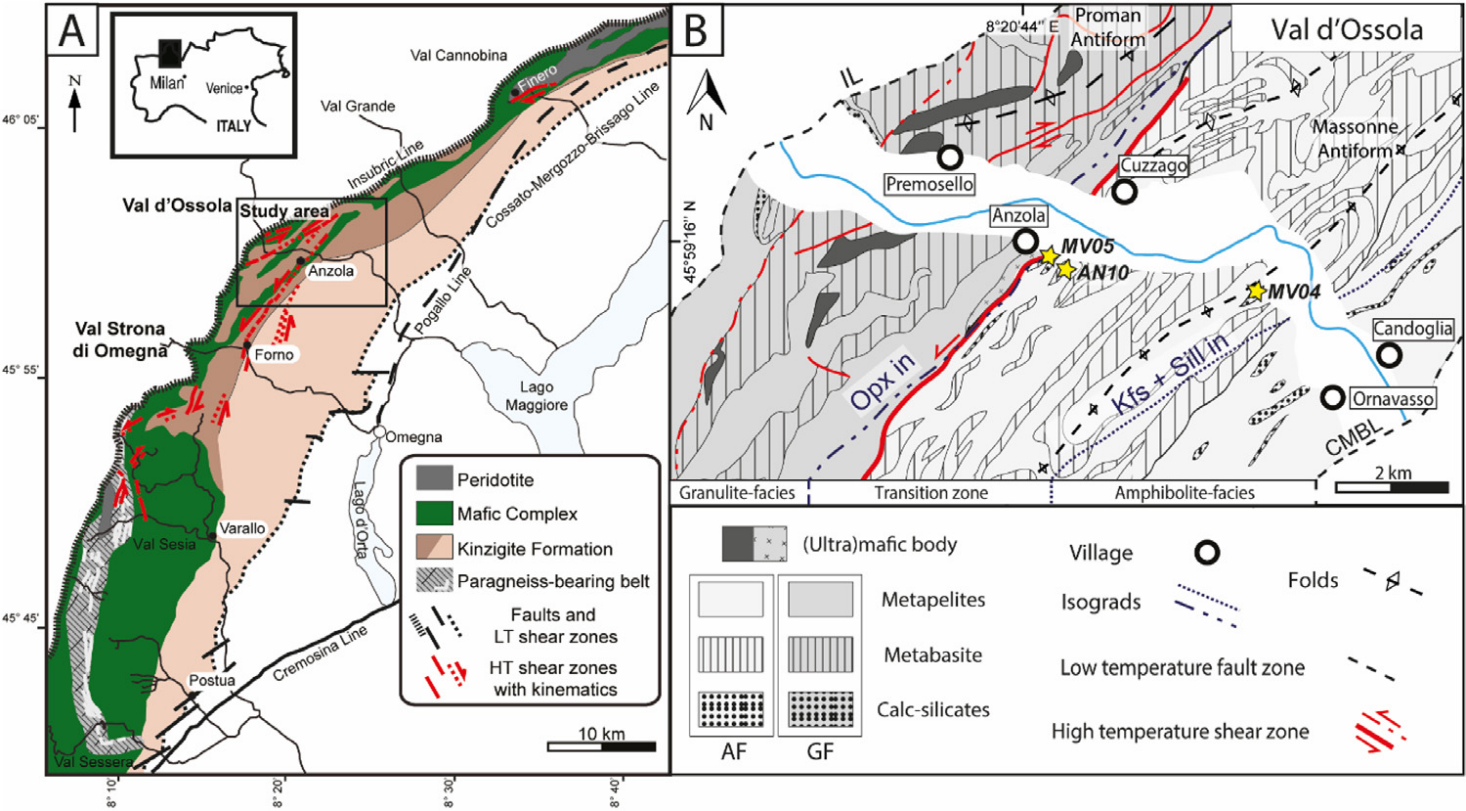
A) Geological sketch map of the Ivrea-Verbano Zone, modified after [7]. B) Schematic geological map showing the main lithologies, tectonic structures, isograds and samples location through the Val d'Ossola transect (modified after [1]).

The studied titanite-bearing samples crop out in the Ossola valley (north-western Italy) where the Ivrea-Verbano Zone (IVZ) displays one of the best-preserved cross sections through the middle to lower Variscan continental crust (Fig. 1A; [1]). The IVZ consists of a pre-Variscan volcano-sedimentary metamorphic sequence (i.e., Kinzigite Formation) made up of metapelites, amphibolite and marbles overlaying minor peridotitic bodies (i.e., Finero, Balmuccia, Premosello, Baldissero), gabbros and diorites (i.e., Mafic Complex; Fig. 1; [8,9,10]).

The IVZ consists of a pre-Variscan volcano-sedimentary metamorphic sequence (i.e., Kinzigite Formation) made up of metapelites, amphibolite and marbles overlaying minor peridotitic bodies (i.e., Finero, Balmuccia, Premosello, Baldissero), gabbros and diorites (i.e., Mafic Complex; Fig. 1; [[8], [9], [10]]). The crustal emplacement of peridotitic bodies in different stratigraphic levels of the crust is considered a consequence of the tectonic evolution of the Kinzigite Formation that occurred in the end of the Variscan orogeny and before the intrusion of the Mafic Complex [8,9]. The Mafic Complex, variably overprinted by deformation and metamorphism, intruded into the metasedimentary sequences of the Kinzigite Formation. In particular, in the south-western part of the IVZ, the transition between the upper and lower Mafic Complex corresponds to a zone, identified as the “paragneiss-bearing belt”, where paragneiss septa (i.e., depleted granulite) are interlayered with igneous rocks (Fig. 1A; [8,9]). Mantle-derived mafic magmatism started in the Carboniferous (∼314; [9]), mainly developed during the early Permian (290–270 Ma; [11]) and locally occurred up to the Triassic-Jurassic [12 and reference therein]. At a regional scale, peak metamorphic grade decreases from granulite (∼900 °C; 0.9 GPa) to amphibolite facies (∼600 °C; ∼0.4 GPa) from NW to SE [8,10]. Long-lasting high-grade metamorphism developed between the Late Carboniferous (∼316 Ma) and the Early Permian, coeval with the Mafic Complex intrusion [13].

The spatially progressive switch from granulite to amphibolite facies is marked by a transition zone characterized by abundant migmatites that were involved in several high-temperature (granulite-amphibolite facies) mylonitic shear zones, including the Anzola shear zone (Fig. 1B; [1,6,7]). These structures are thought to have accommodated, since the Triassic, crustal thinning in the mid-low crust during the early Tethyan rifting [14] and reference therein. At the same time, brittle-ductile shear zones and faults developed in the upper crust (e.g., the Pogallo Line, PL).

Both the activity of the Pogallo Line and the Anzola shear zone is constrained between Triassic and Jurassic times through Ar-Ar and K-Ar dating on micas [7].

### 3.2 Sampling strategy and petrography

**Fig. 2:**
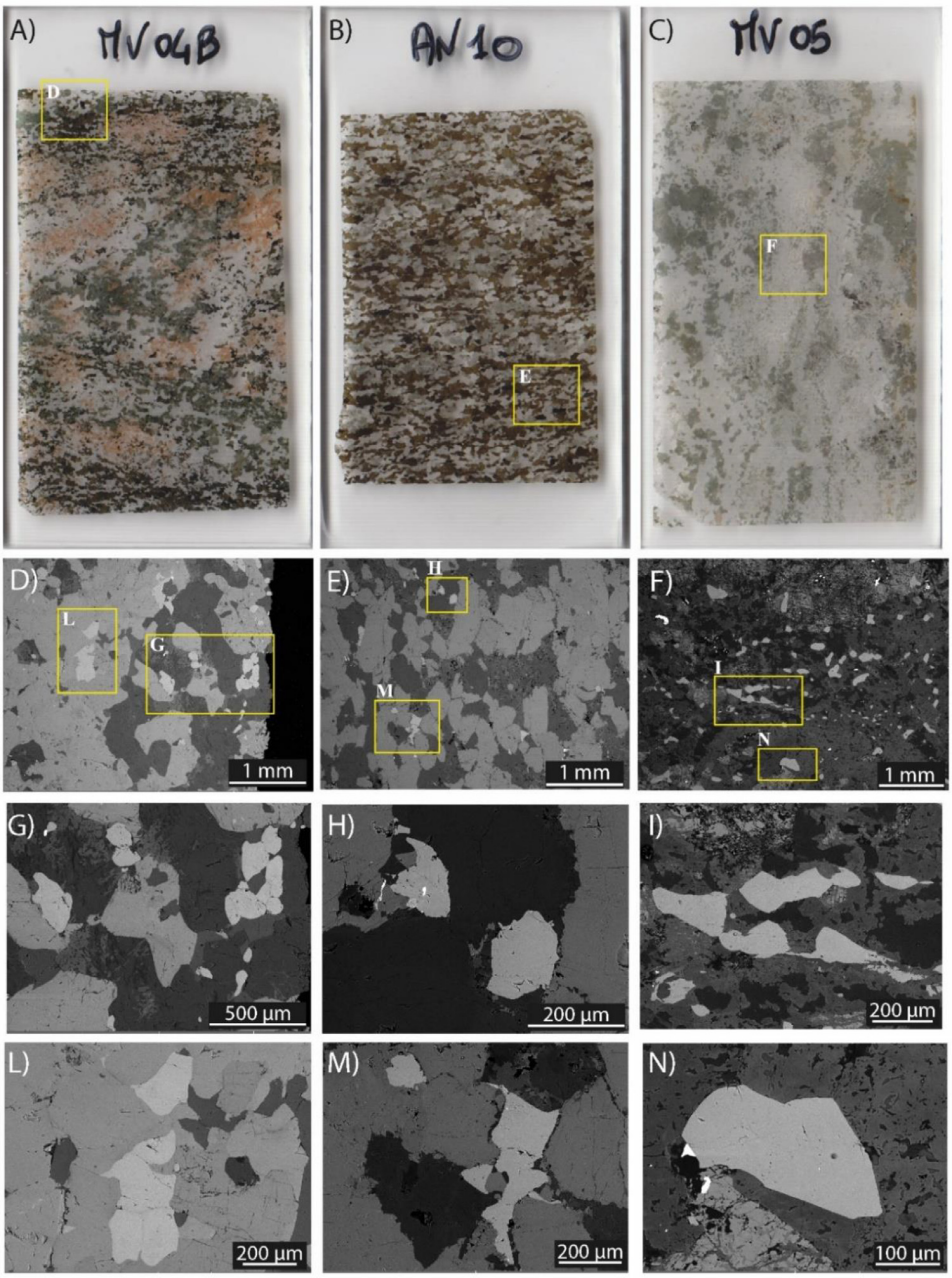
A, B, C) thin section scans of studied samples; C, D, E) BSE image of the titanite locations and textures within the different samples; F, G, H, I, L, M) Detailed BSE images of titanite showing the main textural features.

Petrochronological analyses were performed for titanite from one calc-silicate (MV04B) showing amphibolite-facies conditions and mafic gneiss (AN10) and another calc-silicate sample (MV05) equilibrated at upper amphibolites- to granulite-facies conditions (Fig. 1B; Table 1; for more field and petrographic details see [1]). Amphibolite-facies calc-silicate (MV04B) has a granoblastic texture and consists mostly of plagioclase, green amphibole, pinkish garnet, light green clinopyroxene and calcite (Fig. 2A; Table 1). Mafic gneiss (AN10) shows a nematoblastic texture consisting mainly of brown amphibole and plagioclase with minor light green clinopyroxene and scapolite (Fig. 2B; Table 1). The calc-silicate (MV05) is generally coarse-grained (mm-cm sized) with a granoblastic texture, and the mineral assemblage is mainly composed by plagioclase, K-feldspar, clinopyroxene, calcite, scapolite, and epidote (Fig. 2C; Table 1). In all samples, oxides are common, and zircon and apatite are other accessory minerals. Titanite grains occur dispersed in the matrix as sigmoidal/lozenge shaped crystals of about 250×500 µm, locally forming aggregates of grains (Fig. 2D-N).

**Table 1:** List of locality, coordinates and mineral assemblages of the studied samples from Val d'Ossola transect from SE to NW. Mineral abbreviations are after [15].

Sample	Locality	Lithology	Coordinates		Mineral
			E	N	assemblage
**MV04B**	Ornavasso	Calc-silicate	8° 23′ 48″	45° 58′ 56″	Pl + Grt + Px + Amph + Cal + Ttn
**AN10**	Anzola	Mafic gneiss	8° 20′ 47″	45° 59′ 10″	Pl + Cpx + Amph + Scp + Ttn
**MV05**	Anzola	Calc-silicate	8° 21′ 08″	45° 59′ 08″	Pl + Kfs + Cpx + Amph + Cal + Scp+ Ep + Ttn

### 3.3 U–Pb LASS-ICP-MS petrochronology

#### 3.3.1 Calc-silicate (MV04B) – Ornavasso – amphibolite-facies

**Fig. 3:**
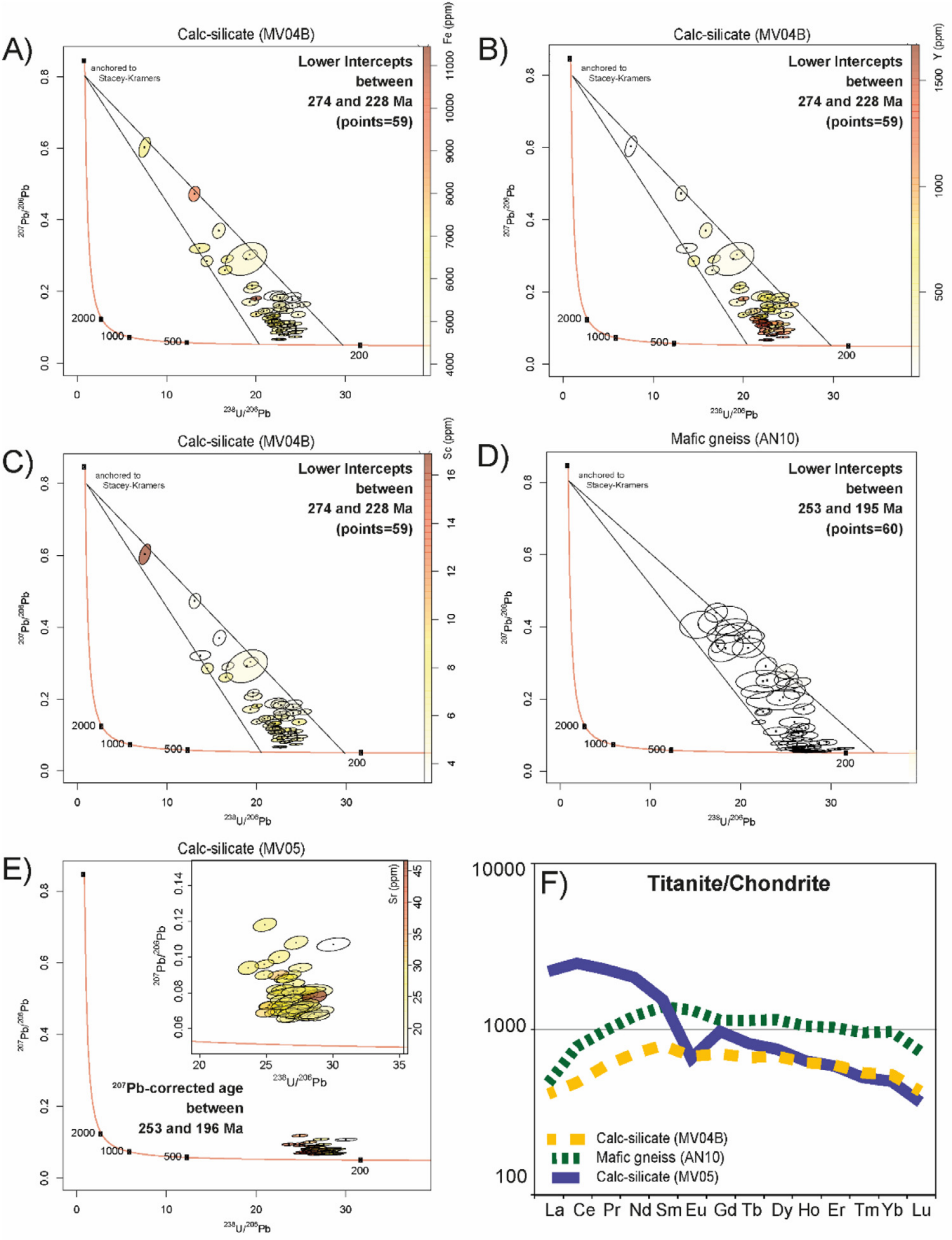
Tera-Wasserburg concordia diagrams reporting representative correlations between titanite U–Pb data and Fe (A), Y (B), Sc (C) for the amphibolite-facies MV04B calc-silicate, D) Tera-Wasserburg concordia diagram for the upper amphibolite- to granulite-facies mafic gneiss (AN10) without correlation with chemistry; E) Tera-Wasserburg concordia diagram for the upper amphibolite- to granulite-facies calc-silicate (MV05) showing the correlation with Sc data. F) Chondrite-normalised REE patterns of titanite. Chondrite values from [17].

A total of 59 in-situ analyses were performed on 27 titanite grains (Table 2). The U–Pb data do not show good alignments and are delimited by lower intercepts at about 274 and 228 Ma (Fig. 3A). For this sample, U–Pb data show a weak correlation with Fe, Y and Sc. The common Pb (Pb_C_) is broadly inversely correlated with Y, whereas Fe and Sc looks lower for the U–Pb data defining younger intercepts (Table 2; Fig. 3A, B, C). The REE pattern show a flattened trend with average concentrations <1000 times CI, a weak depletion in LREE and a slight Eu negative anomaly (Fig. 3F; Table 5).

**Table 2:** LASS U–Pb results for titanite from calc-silicate sample MV04B.

Analyses	Grain	U ppm	Th ppm	^238^U/^206^Pb	2s	^207^Pb/^206^Pb	2s	rho	^208^Pb/^232^Th	2s	207-corr age	2s	Concordance
**MV04B-1**	1	32	11	23.83	0.57	0.100	0.003	0.47	0.028	0.002	249	6	0.57
**MV04B-2**	2	93	18	22.65	0.54	0.067	0.002	0.40	0.025	0.001	273	6	0.81
**MV04B-3**	2	112	15	22.78	0.50	0.066	0.002	0.24	0.026	0.002	272	6	0.80
**MV04B-4**	2	112	35	22.38	0.52	0.067	0.002	0.20	0.019	0.001	276	6	0.80
**MV04B-5**	2	24	10	22.35	0.54	0.114	0.004	0.19	0.031	0.001	260	7	0.52
**MV04B-6**	2	12	5	23.98	0.89	0.185	0.010	0.34	0.044	0.007	219	9	0.35
**MV04B-7**	2	34	16	22.47	0.55	0.095	0.005	0.23	0.024	0.001	266	7	0.61
**MV04B-8**	2	56	25	23.15	0.51	0.079	0.002	0.29	0.019	0.001	263	6	0.70
**MV04B-9**	2	60	14	23.66	0.56	0.083	0.003	0.60	0.026	0.002	256	6	0.67
**MV04B-10**	2	16	3	20.03	0.52	0.137	0.006	0.27	0.105	0.037	281	8	0.46
**MV04B-11**	3	32	18	24.51	0.97	0.094	0.004	0.44	0.020	0.001	244	10	0.60
**MV04B-12**	4	10	4	22.08	0.98	0.189	0.011	0.29	0.058	0.028	236	12	0.35
**MV04B-13**	5	43	20	22.32	0.57	0.086	0.004	0.23	0.023	0.001	271	7	0.66
**MV04B-14**	5	36	18	22.23	0.60	0.106	0.005	0.28	0.026	0.001	264	7	0.54
**MV04B-15**	5	31	13	21.73	0.56	0.121	0.005	0.29	0.031	0.002	265	7	0.50
**MV04B-16**	5	57	27	22.43	0.51	0.113	0.003	0.18	0.028	0.001	260	6	0.52
**MV04B-17**	5	15	6	22.83	0.73	0.155	0.009	0.16	0.047	0.009	240	9	0.42
**MV04B-18**	6	28	10	22.11	0.53	0.119	0.005	0.24	0.034	0.002	261	7	0.50
**MV04B-19**	6	33	12	22.06	0.57	0.117	0.005	0.23	0.035	0.002	262	7	0.52
**MV04B-20**	6	13	5	22.38	0.65	0.186	0.010	0.17	0.052	0.009	234	8	0.36
**MV04B-21**	7	16	2	23.42	0.64	0.146	0.006	0.24	0.024	0.069	237	7	0.43
**MV04B-22**	7	8	1	19.61	0.80	0.207	0.009	0.17	0.060	0.140	258	12	0.35
**MV04B-24**	7	16	6	22.79	0.61	0.152	0.006	0.16	0.045	0.005	242	7	0.42
**MV04B-25**	8	77	27	24.39	0.60	0.074	0.002	0.27	0.019	0.001	252	6	0.73
**MV04B-26**	9	43	17	24.50	0.59	0.087	0.004	0.12	0.022	0.001	246	6	0.63
**MV04B-27**	9	11	1	19.34	0.74	0.171	0.008	0.08	0.140	0.120	277	11	0.40
**MV04B-28**	9	29	3	24.39	0.62	0.110	0.004	0.27	0.060	0.043	240	6	0.53
**MV04B-29**	10	11	5	19.34	0.65	0.303	0.011	0.28	0.101	0.023	222	11	0.28
**MV04B-30**	11	10	4	13.12	0.52	0.473	0.017	0.20	0.245	0.041	223	21	0.26
**MV04B-31**	12	14	5	24.08	0.73	0.178	0.007	0.17	0.055	0.017	220	7	0.37
**MV04B-32**	12	14	2	22.73	0.73	0.183	0.008	0.14	0.140	0.180	232	8	0.36
**MV04B-33**	12	22	2	25.36	0.61	0.164	0.006	0.26	0.153	0.073	214	6	0.39
**MV04B-34**	12	45	16	23.74	0.56	0.114	0.005	0.20	0.031	0.001	245	6	0.52
**MV04B-35**	12	9	1	14.51	0.53	0.285	0.012	0.12	0.400	0.590	305	15	0.32
**MV04B-36**	12	38	18	21.25	0.55	0.147	0.006	0.26	0.039	0.002	261	7	0.44
**MV04B-37**	13	39	19	20.02	0.49	0.181	0.005	0.26	0.047	0.002	263	7	0.38
**MV04B-38**	13	23	10	20.89	0.55	0.145	0.007	0.12	0.038	0.003	266	8	0.44
**MV04B-39**	14	18	2	24.07	0.71	0.161	0.006	0.24	0.190	0.110	226	7	0.39
**MV04B-40**	15	28	11	23.09	0.63	0.113	0.005	0.17	0.029	0.002	252	7	0.52
**MV04B-41**	16	26	9	22.63	0.61	0.163	0.007	0.15	0.051	0.004	240	7	0.40
**MV04B-42**	17	28	10	21.73	0.58	0.133	0.005	0.23	0.039	0.003	261	7	0.47
**MV04B-43**	18	29	9	16.78	0.56	0.291	0.009	0.35	0.130	0.008	261	12	0.30
**MV04B-44**	19	13	2	15.85	0.57	0.370	0.017	0.25	0.460	0.160	237	16	0.27
**MV04B-45**	20	16	4	21.83	0.72	0.135	0.006	0.17	0.059	0.015	259	9	0.46
**MV04B-46**	21	6	2	7.54	0.53	0.603	0.023	0.46	0.730	0.870	249	48	0.32
**MV04B-47**	21	9	3	18.90	1.86	0.291	0.037	0.29	0.106	0.044	232	28	0.28
**MV04B-48**	22	39	11	23.57	0.55	0.105	0.004	0.30	0.032	0.002	250	6	0.55
**MV04B-49**	22	5	1	13.72	0.93	0.321	0.011	0.19	0.380	0.240	302	23	0.31
**MV04B-50**	22	25	8	19.62	0.55	0.217	0.008	0.29	0.081	0.010	254	9	0.34
**MV04B-51**	23	19	7	24.73	0.65	0.136	0.007	0.13	0.035	0.003	228	7	0.44
**MV04B-52**	23	21	6	21.95	0.57	0.131	0.005	0.41	0.049	0.005	258	7	0.47
**MV04B-53**	23	11	4	16.56	0.62	0.260	0.010	0.21	0.114	0.021	280	13	0.32
**MV04B-54**	24	41	11	22.83	0.53	0.092	0.004	0.28	0.030	0.002	262	6	0.62
**MV04B-55**	24	26	10	22.11	0.53	0.132	0.005	0.16	0.035	0.002	256	7	0.47
**MV04B-56**	25	35	22	22.48	0.59	0.111	0.004	0.13	0.022	0.001	260	7	0.53
**MV04B-57**	25	31	18	22.41	0.62	0.108	0.003	0.14	0.024	0.001	261	7	0.54
**MV04B-58**	26	28	14	21.79	0.64	0.117	0.004	0.29	0.027	0.002	266	8	0.51
**MV04B-59**	26	31	16	21.30	0.51	0.126	0.005	0.15	0.030	0.001	268	7	0.49
**MV04B-60**	27	44	35	22.03	0.61	0.108	0.004	0.29	0.021	0.001	266	7	0.54

#### 3.3.2 Mafic gneiss (AN10) – anzola – upper amphibolite- to granulite-facies

A total of 60 analyses were performed on 23 grains (Table 3). The U–Pb data do not show any correlation with chemistry and textural position. All the isotopic ratios are delimited by lower intercepts at about 253 and 195 Ma (Fig. 3D). The REE pattern mostly mimic the REE pattern of calc-silicate (MV04B) at higher concentration, exceeding 1000 times CI in most cases (Fig. 3F; Table 6).

**Table 3:** LASS U–Pb results for titanite from mafic gneiss sample AN10.

Analyses	Grain	U ppm	Th ppm	^238^U/^206^Pb	2s	^207^Pb/^206^Pb	2s	rho	^208^Pb/^232^Th	2s	207-corr age	2s	Concordance
**AN10-1**	1	4	3	21.83	1.45	0.220	0.015	0.14	0.046	0.010	228	16	0.32
**AN10-2**	1	3	2	15.34	1.70	0.405	0.030	0.11	0.010	0.140	227	32	0.27
**AN10-3**	2	3	2	17.99	2.20	0.417	0.032	0.20	0.100	0.160	188	29	0.23
**AN10-4**	2	3	2	24.33	3.00	0.196	0.021	0.07	0.046	0.024	212	27	0.34
**AN10-5**	3	64	32	26.39	0.63	0.064	0.003	0.32	0.015	0.001	236	6	0.84
**AN10-6**	3	112	61	26.92	0.59	0.054	0.002	0.25	0.012	0.000	234	5	0.94
**AN10-7**	4	7	5	18.35	1.56	0.340	0.030	0.30	0.068	0.014	218	24	0.26
**AN10-8**	4	10	8	27.20	0.85	0.119	0.010	0.09	0.018	0.002	213	7	0.48
**AN10-9**	4	13	7	25.84	0.95	0.089	0.007	0.10	0.017	0.002	233	9	0.63
**AN10-10**	5	3	3	19.19	1.63	0.398	0.019	0.13	0.075	0.021	184	20	0.24
**AN10-11**	5	13	6	22.78	0.95	0.290	0.020	0.27	0.067	0.010	194	12	0.27
**AN10-12**	6	12	7	26.53	0.85	0.121	0.009	0.12	0.023	0.002	218	8	0.48
**AN10-13**	7	5	6	25.97	0.96	0.221	0.017	0.20	0.027	0.004	191	9	0.31
**AN10-14**	7	5	5	17.27	1.02	0.439	0.021	0.13	0.078	0.010	186	19	0.23
**AN10-15**	8	89	44	30.43	0.68	0.060	0.002	0.18	0.012	0.000	206	5	0.86
**AN10-16**	8	99	52	28.51	0.66	0.057	0.002	0.12	0.012	0.000	221	5	0.91
**AN10-17**	9	3	3	19.76	2.07	0.366	0.036	0.09	0.049	0.043	192	26	0.24
**AN10-18**	9	5	4	24.69	1.49	0.204	0.018	0.12	0.037	0.012	207	14	0.34
**AN10-19**	10	4	3	20.83	1.45	0.341	0.022	0.11	0.061	0.025	192	17	0.25
**AN10-20**	11	14	14	26.81	0.90	0.086	0.005	0.13	0.014	0.001	226	8	0.65
**AN10-21**	11	15	11	28.72	0.92	0.078	0.005	0.08	0.014	0.001	213	7	0.68
**AN10-22**	11	11	8	27.03	0.97	0.172	0.012	0.07	0.024	0.002	198	8	0.37
**AN10-23**	12	33	20	27.47	0.65	0.065	0.004	0.29	0.013	0.000	226	5	0.81
**AN10-24**	12	33	19	25.94	0.70	0.112	0.006	0.39	0.021	0.001	225	6	0.51
**AN10-25**	13	14	15	29.56	0.90	0.080	0.007	0.08	0.011	0.001	207	7	0.67
**AN10-26**	13	4	2	20.96	1.34	0.372	0.015	0.13	0.105	0.032	179	15	0.24
**AN10-27**	14	95	40	28.06	0.61	0.055	0.002	0.15	0.012	0.000	225	5	0.93
**AN10-28**	14	134	65	26.67	0.66	0.055	0.002	0.44	0.012	0.000	236	6	0.94
**AN10-29**	14	139	82	27.41	0.60	0.053	0.002	0.20	0.012	0.000	230	5	0.96
**AN10-30**	14	84	34	27.23	0.59	0.056	0.002	0.17	0.012	0.000	231	5	0.92
**AN10-31**	14	90	40	28.20	0.62	0.070	0.002	0.14	0.014	0.000	219	5	0.75
**AN10-32**	14	18	15	26.46	0.72	0.105	0.006	0.12	0.016	0.001	223	6	0.54
**AN10-33**	14	98	45	26.27	0.56	0.056	0.002	0.23	0.012	0.000	239	5	0.93
**AN10-34**	14	114	57	26.46	0.59	0.054	0.002	0.13	0.012	0.000	238	5	0.94
**AN10-35**	14	145	83	28.17	0.59	0.065	0.002	0.17	0.013	0.000	221	5	0.80
**AN10-36**	15	5	3	25.06	1.07	0.255	0.016	0.18	0.054	0.017	187	10	0.28
**AN10-37**	15	6	5	22.94	1.01	0.251	0.016	0.11	0.041	0.008	206	11	0.29
**AN10-38**	15	10	7	25.38	1.15	0.241	0.012	0.19	0.035	0.004	189	10	0.30
**AN10-39**	15	10	10	25.06	0.96	0.276	0.016	0.16	0.031	0.003	181	10	0.27
**AN10-40**	15	12	11	26.39	1.11	0.245	0.011	0.38	0.030	0.002	181	9	0.28
**AN10-41**	16	31	13	26.20	0.80	0.066	0.003	0.24	0.017	0.001	237	7	0.81
**AN10-42**	16	23	8	22.00	0.62	0.135	0.005	0.29	0.043	0.004	257	8	0.46
**AN10-43**	17	100	49	31.56	0.84	0.063	0.002	0.23	0.013	0.000	198	5	0.81
**AN10-44**	17	135	68	28.94	0.90	0.054	0.002	0.33	0.012	0.000	218	7	0.94
**AN10-45**	17	136	73	28.46	0.80	0.054	0.002	0.47	0.012	0.000	222	6	0.95
**AN10-46**	18	57	29	26.53	0.94	0.058	0.002	0.37	0.014	0.001	237	8	0.89
**AN10-47**	19	17	10	26.25	0.92	0.072	0.004	0.28	0.017	0.001	235	8	0.72
**AN10-48**	19	41	14	26.03	0.77	0.061	0.003	0.32	0.017	0.001	240	7	0.85
**AN10-49**	19	39	14	25.25	0.86	0.078	0.004	0.29	0.021	0.001	242	8	0.70
**AN10-50**	19	36	13	22.47	0.61	0.164	0.006	0.28	0.050	0.004	241	7	0.40
**AN10-51**	20	9	4	25.64	1.05	0.100	0.007	0.36	0.036	0.009	231	10	0.55
**AN10-52**	20	36	16	25.00	0.85	0.061	0.003	0.24	0.017	0.001	250	8	0.86
**AN10-53**	20	30	10	24.24	0.75	0.077	0.005	0.26	0.024	0.002	252	8	0.71
**AN10-54**	20	3	1	22.47	2.42	0.249	0.024	0.08	0.015	0.025	211	24	0.30
**AN10-55**	21	7	5	26.81	1.40	0.097	0.010	0.19	0.019	0.003	222	12	0.56
**AN10-56**	22	13	8	17.48	0.60	0.346	0.014	0.28	0.076	0.005	226	13	0.27
**AN10-57**	23	13	7	22.52	0.76	0.164	0.009	0.25	0.038	0.005	240	9	0.39
**AN10-58**	23	10	6	23.92	1.14	0.110	0.009	0.23	0.028	0.004	245	12	0.54
**AN10-59**	23	7	7	25.97	1.38	0.136	0.011	0.15	0.021	0.002	217	12	0.44
**AN10-60**	23	4	3	18.38	1.70	0.380	0.021	0.20	0.110	0.057	200	22	0.25
													

#### 3.3.3 Calc-silicate (MV05) - Anzola – upper amphibolite- to granulite-facies

A total of 60 analyses were performed on 17 grains (Table 4). The U–Pb data define a cluster close to the Concordia curve without apparent alignments (Fig. 3E). However, the ^207^Pb-corrected age spread from 253 to 196 Ma (Table 4). Also, for this sample we do not observe significant correlations between U–Pb data and textural position (core vs rims). We rather observed a correlation between U–Pb data and Sr concentrations (Fig. 3E). The REE pattern shows a strong fractionation of LREE (>1000 times CI) over HREE (<1000 times CI) and an apparent Eu negative anomaly (Fig. 3F; Table 7).

**Table 4:** LASS U–Pb results for titanite from calc-silicate sample MV05.

Analyses	Grain	U ppm	Th ppm	^238^U/^206^Pb	2s	^207^Pb/^206^Pb	2s	rho	^208^Pb/^232^Th	2s	207-corr age	2s	Concordance
**MV05-1**	1	257	521	27.65	0.69	0.078	0.002	0.24	0.012	0.000	221	6	0.69
**MV05-2**	1	246	386	26.94	0.65	0.070	0.002	0.24	0.012	0.000	229	6	0.75
**MV05-3**	1	234	347	25.83	0.59	0.073	0.002	0.28	0.013	0.000	238	5	0.73
**MV05-4**	1	214	271	27.55	0.71	0.094	0.002	0.27	0.013	0.000	217	6	0.59
**MV05-5**	1	228	345	26.32	0.59	0.088	0.002	0.25	0.013	0.000	229	5	0.62
**MV05-6**	1	234	359	26.05	0.60	0.072	0.002	0.25	0.013	0.000	236	5	0.75
**MV05-7**	1	257	574	27.83	0.83	0.081	0.002	0.20	0.012	0.000	219	6	0.68
**MV05-8**	2	242	504	25.95	0.65	0.100	0.003	0.33	0.014	0.000	229	6	0.57
**MV05-9**	2	208	379	25.73	0.59	0.082	0.002	0.24	0.013	0.000	236	5	0.67
**MV05-10**	2	281	493	25.22	0.64	0.073	0.002	0.36	0.013	0.000	244	6	0.74
**MV05-11**	3	273	566	27.92	0.75	0.079	0.002	0.22	0.012	0.000	219	6	0.68
**MV05-12**	3	267	531	26.89	0.59	0.090	0.002	0.37	0.013	0.000	224	5	0.61
**MV05-13**	3	269	565	25.90	0.62	0.090	0.002	0.34	0.014	0.000	232	6	0.62
**MV05-14**	3	257	684	24.83	0.55	0.090	0.002	0.27	0.014	0.000	242	5	0.62
**MV05-15**	4	250	558	24.86	0.58	0.096	0.002	0.37	0.014	0.000	240	6	0.59
**MV05-16**	4	262	483	27.04	0.67	0.085	0.002	0.36	0.013	0.000	224	6	0.63
**MV05-17**	4	265	581	26.25	0.70	0.082	0.002	0.33	0.013	0.000	232	6	0.66
**MV05-18**	5	263	507	27.38	0.68	0.079	0.002	0.20	0.012	0.000	223	6	0.68
**MV05-19**	6	249	590	25.87	0.67	0.079	0.002	0.43	0.013	0.000	236	6	0.69
**MV05-20**	6	261	560	27.59	0.59	0.077	0.002	0.32	0.012	0.000	222	5	0.70
**MV05-21**	7	231	360	27.36	0.77	0.068	0.002	0.49	0.012	0.000	226	6	0.79
**MV05-22**	7	219	362	25.27	0.64	0.073	0.002	0.36	0.013	0.000	243	6	0.73
**MV05-23**	8	252	420	26.00	0.57	0.076	0.002	0.15	0.013	0.000	236	5	0.71
**MV05-24**	8	241	328	27.59	0.63	0.073	0.002	0.43	0.013	0.000	223	5	0.72
**MV05-25**	7	228	293	25.87	0.62	0.070	0.002	0.37	0.014	0.000	239	6	0.77
**MV05-26**	7	215	163	25.65	0.62	0.069	0.002	0.31	0.014	0.000	241	6	0.77
**MV05-27**	7	202	175	26.49	0.61	0.070	0.002	0.26	0.014	0.000	233	5	0.76
**MV05-28**	7	206	306	26.08	0.64	0.070	0.002	0.33	0.013	0.000	237	6	0.76
**MV05-29**	7	229	559	26.85	0.68	0.066	0.002	0.30	0.012	0.000	231	6	0.80
**MV05-30**	7	262	419	26.49	0.59	0.066	0.002	0.26	0.012	0.000	234	5	0.80
**MV05-31**	9	249	463	28.57	1.13	0.081	0.003	0.13	0.012	0.000	213	8	0.66
**MV05-32**	9	235	399	27.21	0.62	0.068	0.002	0.39	0.013	0.000	228	5	0.77
**MV05-33**	9	232	390	28.04	0.75	0.067	0.002	0.31	0.012	0.000	221	6	0.78
**MV05-34**	9	232	403	28.30	0.68	0.069	0.002	0.39	0.012	0.000	219	5	0.76
**MV05-35**	9	242	412	28.32	0.63	0.067	0.002	0.24	0.012	0.000	219	5	0.79
**MV05-36**	9	239	435	27.49	0.71	0.072	0.002	0.29	0.012	0.000	224	6	0.74
**MV05-37**	9	239	496	29.21	0.69	0.070	0.002	0.25	0.011	0.000	212	5	0.75
**MV05-38**	9	217	381	26.55	0.71	0.075	0.002	0.30	0.013	0.000	231	6	0.71
**MV05-39**	9	216	326	26.80	0.63	0.071	0.002	0.26	0.013	0.000	230	5	0.76
**MV05-40**	9	242	431	27.44	0.63	0.069	0.002	0.28	0.012	0.000	225	5	0.76
**MV05-41**	10	233	554	28.60	0.73	0.078	0.003	0.19	0.012	0.000	214	5	0.69
**MV05-42**	10	251	572	28.22	0.72	0.072	0.002	0.31	0.012	0.000	218	6	0.74
**MV05-43**	10	256	586	28.28	0.72	0.067	0.002	0.33	0.012	0.000	219	6	0.78
**MV05-44**	10	257	557	28.91	0.68	0.067	0.002	0.35	0.011	0.000	215	5	0.77
**MV05-45**	11	220	469	24.91	0.58	0.069	0.002	0.16	0.013	0.000	248	6	0.77
**MV05-46**	12	248	693	26.55	0.63	0.088	0.002	0.40	0.012	0.000	227	5	0.63
**MV05-47**	12	228	471	27.92	0.73	0.081	0.002	0.29	0.013	0.000	218	6	0.67
**MV05-48**	12	177	420	27.32	1.73	0.081	0.002	0.51	0.012	0.001	223	14	0.68
**MV05-49**	12	230	601	28.15	0.65	0.074	0.002	0.23	0.012	0.000	218	5	0.71
**MV05-50**	12	234	921	26.20	0.65	0.073	0.002	0.27	0.012	0.000	235	6	0.75
**MV05-51**	13	238	363	27.23	0.72	0.108	0.003	0.37	0.015	0.000	216	6	0.52
**MV05-52**	13	188	366	24.89	0.71	0.118	0.003	0.31	0.016	0.000	233	7	0.50
**MV05-53**	13	229	304	27.69	0.70	0.073	0.002	0.40	0.014	0.000	222	6	0.73
**MV05-54**	13	255	255	28.60	0.87	0.068	0.002	0.51	0.013	0.000	217	7	0.78
**MV05-55**	13	234	211	27.03	0.73	0.081	0.002	0.37	0.016	0.000	225	6	0.67
**MV05-56**	13	225	375	26.72	0.68	0.069	0.002	0.39	0.013	0.000	231	6	0.76
**MV05-57**	14	112	67	23.65	0.62	0.094	0.003	0.24	0.021	0.001	253	7	0.60
**MV05-58**	15	241	410	27.34	0.72	0.071	0.002	0.37	0.013	0.000	226	6	0.74
**MV05-59**	16	254	317	26.86	0.68	0.077	0.002	0.49	0.014	0.000	228	6	0.69
**MV05-60**	17	255	281	30.03	1.00	0.107	0.003	0.33	0.015	0.000	196	7	0.52

**Table 5:** LASS trace element concentration results for titanite from calc-silicate sample MV04.

**Table 6:** LASS trace element concentration results for titanite from mafic gneiss sample AN10.

**Table 7:** LASS trace element concentration results for titanite from calc-silicate sample MV05.

### 3.4 Origin of titanite

**Fig. 4:**
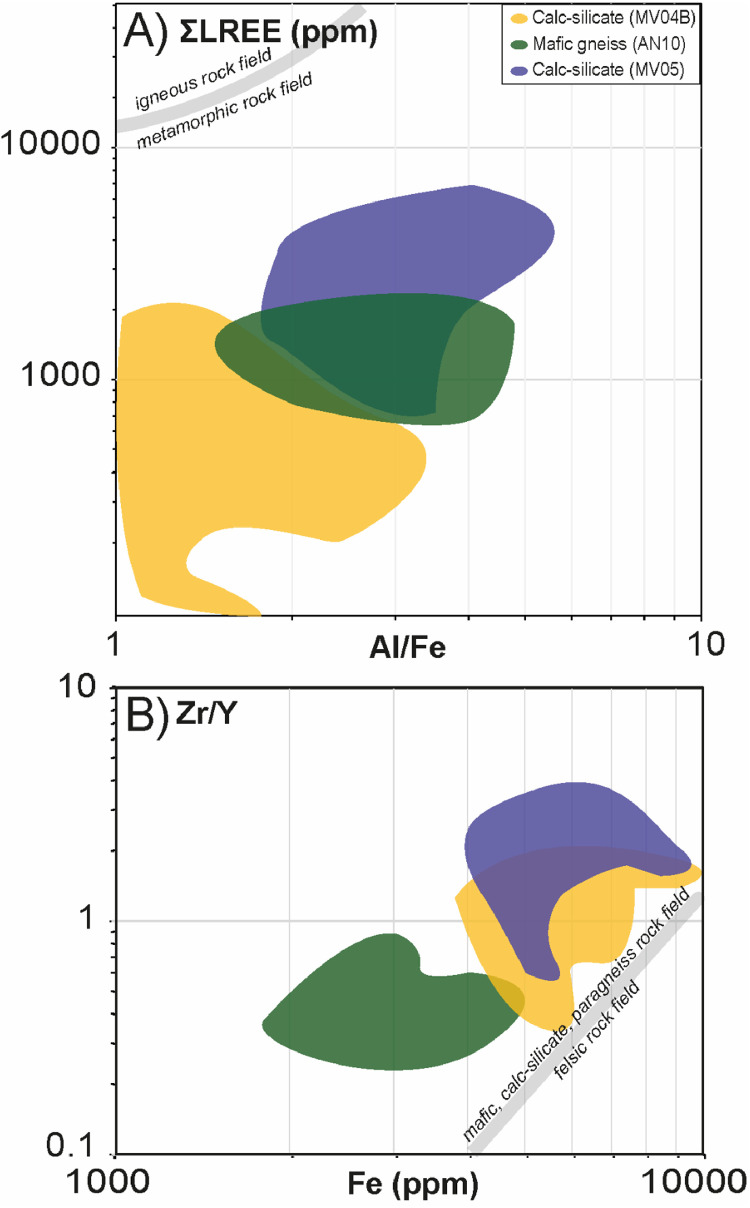
Representative trace-elements concentrations diagrams of titanite from calc-silicates and mafic gneiss (see the legend for details). A) Al/Fe versus ∑LREE cross plot. B) Fe (ppm) versus Zr/Y cross plot. Data are represented as cloud and colored differently to distinguish the samples.

In order to discriminate the possible origin of the studied titanite, we plotted Al/Fe versus ΣLREE (Fig. 4A) and Fe content versus Zr/Y (Fig. 4B) according to [16]. Although titanite grains from the three studied samples define distinct clusters on the Al/Fe versus ΣLREE diagram, they fall in the field of metamorphic titanite (Al/Fe =1–10; ΣLREE =100–10,000; Fig. 4A). The Fe versus Zr/Y diagram (Fig. 4B) shows that titanite from the calc-silicates (MV04, MV05) and mafic gneiss (AN10) is compatible with mafic (i.e., amphibolites) and calc-silicate protoliths (Fe =1000–10,000; Zr/*Y* = 1 for calc-silicates, Zr/*Y* = 0.1–1 for mafic gneiss), as previously demonstrated on the basis geochemistry and petrography [1].

## 4 Experimental Design, Materials and Methods

### 4.1 Material and methods

Titanite grains from studied samples were first selected after preliminary observations of polished thin sections under a polarization microscope and then producing detailed images by using a scanning electron microscope (SEM-BSE) Tescan Mira3 XMU-series FESEM equipped with an EDAX-EDX at the “Arvedi” laboratory at the University of Pavia.

#### 4.1.1 *U–Pb isotopic and trace-element data using LASS-ICP-MS* analysis

U–Pb isotopic concentrations and trace-element compositions in titanite grains were collected simultaneously from the same spot using Laser ablation split-stream (LASS) technique at the University of California Santa Barbara following methods of [18,19]. Instrumentation consists of a Photon Machines 193 nm ArF Excimer laser and ‘HelEx’ ablation cell coupled to a Nu Instruments HR Plasma high-resolution ICPMS (U, Th, and Pb isotopes) and an Agilent 7700X Quadrupole ICP-MS (major and trace-elements). Typical laser analyses were run with a 50 μm diameter laser spot at 5 Hz for 60 shots, resulting in pits that are ∼5 μm deep. Spot location was guided with the aid of BSE images.

Titanite U–Pb data were determined assuming the Stacey and Kramers (1975) common^207^Pb/^206^Pb ratio of 0.83 ± 0.04, which matches the upper Tera-Wasserburg concordia intercept defined by the distribution of U–Pb rations in the analysed titanite. The ^238^U/^206^Pb and ^207^Pb/^206^Pb isotopic ratios for each analysis were plotted on Tera– Wasserburg concordia diagrams using IsoplotR [20]. All date uncertainties are reported at the 95 % confidence interval, assuming a Gaussian distribution of measurement errors. Because the samples in this study exhibit a broad spread of U/Pb ratios—and thus, well-constrained common ^207^Pb/^206^Pb ratios—we report the ^207^Pb-corrected ^206^Pb/^238^U date obtained by regressing ^238^U/^206^Pb vs ^207^Pb/^206^Pb. Stated 2σ date uncertainties are internal; that is, they include in-run errors and decay constant errors only.

## Limitations

None.

## Ethics Statement

The authors declare that they have read and follow the ethical requirements for publication in Data in Brief and confirm that the current work does not involves human subjects, animal experiments, or any data collected from social media platforms.

## CRediT Author Statement

**Stefania Corvò:** Conceptualization, Investigation, Methodology, Validation, Formal analysis, Writing - original draft, Visualization. **Andrew Kylander-Clark:** Methodology, Validation, Data Curation, Writing - review & editing. **Antonio Langone:** Conceptualization, Investigation, Methodology, Validation, Data Curation, Writing - review & editing, Supervision, Funding acquisition.

## Data Availability

Corvò et al. (2024) Data in Brief_U-Pb data by LASS method on Titanite (Original data) (Mendeley Data)
